# Yoghurt Intake and Gastric Cancer: A Pooled Analysis of 16 Studies of the StoP Consortium

**DOI:** 10.3390/nu15081877

**Published:** 2023-04-13

**Authors:** Giulia Collatuzzo, Eva Negri, Claudio Pelucchi, Rossella Bonzi, Federica Turati, Charles S. Rabkin, Linda M. Liao, Rashmi Sinha, Domenico Palli, Monica Ferraroni, Lizbeth López-Carrillo, Nuno Lunet, Samantha Morais, Demetrius Albanes, Stephanie J. Weinstein, Dominick Parisi, David Zaridze, Dmitry Maximovitch, Trinidad Dierssen-Sotos, José Juan Jiménez-Moleón, Jesus Vioque, Manoli Garcia de la Hera, Maria Paula Curado, Emmanuel Dias-Neto, Raúl Ulises Hernández-Ramírez, Malaquias López-Cervantes, Mary H. Ward, Shoichiro Tsugane, Akihisa Hidaka, Areti Lagiou, Pagona Lagiou, Zuo-Feng Zhang, Antonia Trichopoulou, Anna Karakatsani, Maria Constanza Camargo, Carlo La Vecchia, Paolo Boffetta

**Affiliations:** 1 Department of Medical and Surgical Sciences, University of Bologna, 40138 Bologna, Italy; 2 Department of Clinical Sciences and Community Health, University of Milan, 20122 Milan, Italy; 3 Division of Cancer Epidemiology and Genetics, National Cancer Institute, Rockville, MD 20850, USA; 4 Cancer Risk Factors and Life-Style Epidemiology Unit, Institute for Cancer Research, Prevention and Clinical Network, ISPRO, 50139 Florence, Italy; 5 Mexico National Institute of Public Health, Cuernavaca 62100, Mexico; 6 EPIUnit—Instituto de Saúde Pública da Universidade do Porto, 4050-600 Porto, Portugal; 7 Laboratório para a Investigação Integrativa e Translacional em Saúde Populacional (ITR), 4050-600 Porto, Portugal; 8 Departamento de Ciências da Saúde Pública e Forenses e Educação Médica, Faculdade de Medicina da Universidade do Porto, 4200-450 Porto, Portugal; 9 Information management Services, Inc., Silver Spring, MD 20904, USA; 10 Department of Clinical Epidemiology, N.N. Blokhin National Medical Research Center for Oncology, 115478 Moscow, Russia; 11 Consortium for Biomedical Research in Epidemiology and Public Health (CIBERESP), 28029 Madrid, Spain; 12 Department of Preventive Medicine and Public Health, University of Cantabria—IDIVAL, 39005 Santander, Spain; 13 Departamento de Medicina Preventiva y Salud Pública, Universidad de Granada, 18010 Granada, Spain; 14 Instituto de Investigación Biosanitaria ibs.GRANADA, 18012 Granada, Spain; 15 Instituto de Investigación Sanitaria y Biomédica de Alicante, Universidad Miguel Hernandez (ISABIAL-UMH), 03202 Alicante, Spain; 16 Centro Internacional de Pesquisa, A. C. Camargo Cancer Center, São Paulo 01508-010, Brazil; 17 Department of Biostatistics, Yale School of Public Health, New Haven, CT 06510, USA; 18 Facultad de Medicina, Universidad Nacional Autónoma de México (UNAM), Coyoacán 04510, Mexico; 19 Epidemiology and Prevention Group, Center for Public Health Sciences, National Cancer Center, Tokyo 104-0045, Japan; 20 National Institute of Health and Nutrition, National Institutes of Biomedical Innovation, Health and Nutrition, Tokyo 162-8636, Japan; 21 Department of Public and Community Health, School of Public Health, University of West Attica, 11521 Athens, Greece; 22 Department of Hygiene, Epidemiology and Medical Statistics, School of Medicine, National and Kapodistrian University of Athens, 11527 Athens, Greece; 23 Department of Epidemiology, Harvard T.H. Chan School of Public Health, Boston, MA 02115, USA; 24 Department of Epidemiology, UCLA Fielding School of Public Health, Jonsson Comprehensive Cancer Center, Los Angeles, CA 10833, USA; 25 Hellenic Health Foundation, 11527 Athens, Greece; 26 2nd Pulmonary Medicine Department, Medical School, “ATTIKON” University Hospital, National and Kapodistrian University of Athens, 11527 Haidari, Greece; 27 Stony Brook Cancer Center, Stony Brook University, Stony Brook, NY 11794, USA

**Keywords:** gastric cancer, diet, nutrition, yoghurt

## Abstract

Background: Yoghurt can modify gastrointestinal disease risk, possibly acting on gut microbiota. Our study aimed at exploring the under-investigated association between yoghurt and gastric cancer (GC). Methods: We pooled data from 16 studies from the Stomach Cancer Pooling (StoP) Project. Total yoghurt intake was derived from food frequency questionnaires. We calculated study-specific odds ratios (ORs) of GC and the corresponding 95% confidence intervals (CIs) for increasing categories of yoghurt consumption using univariate and multivariable unconditional logistic regression models. A two-stage analysis, with a meta-analysis of the pooled adjusted data, was conducted. Results: The analysis included 6278 GC cases and 14,181 controls, including 1179 cardia and 3463 non-cardia, 1191 diffuse and 1717 intestinal cases. The overall meta-analysis revealed no association between increasing portions of yoghurt intake (continuous) and GC (OR = 0.98, 95% CI = 0.94–1.02). When restricting to cohort studies, a borderline inverse relationship was found (OR = 0.93, 95% CI = 0.88–0.99). The adjusted and unadjusted OR were 0.92 (95% CI = 0.85–0.99) and 0.78 (95% CI = 0.73–0.84) for any vs. no yoghurt consumption and GC risk. The OR for 1 category of increase in yoghurt intake was 0.96 (95% CI = 0.91–1.02) for cardia, 1.03 (95% CI = 1.00–1.07) for non-cardia, 1.12 (95% CI = 1.07–1.19) for diffuse and 1.02 (95% CI = 0.97–1.06) for intestinal GC. No effect was seen within hospital-based and population-based studies, nor in men or women. Conclusions: We found no association between yoghurt and GC in the main adjusted models, despite sensitivity analyses suggesting a protective effect. Additional studies should further address this association.

## 1. Introduction

According to the 2018 World Cancer Research Fund (WCRF), there is strong evidence that high-salt and salt-preserved foods, overweight/obesity and heavy alcohol consumption increase the risk of gastric cancer (GC), while there is some evidence that consumption of grilled or barbecued meat and fish, consumption of processed meat, low intake of fruit and citrus fruit increase GC risk [1]. Data on the association of GC with yoghurt intake is scarce. One issue is the difficulty in collecting adequate information on yoghurt consumption, which is usually reported among dairy products in general or together with other foods [2].

The anti-cancer properties of yoghurt have been described [3]. A recent meta-analysis assessed a protective effect of yoghurt consumption and cancer overall, with significant associations for colorectal and bladder cancer, but did not consider GC [4]. Another review described a protective effect of increasing dairy products and yoghurt consumption on oesophagal and colorectal cancers, reporting non-significant results for GC [5].

Yoghurt can be beneficial towards gastrointestinal disease by acting positively on the gut microbiota [3], balancing inflammation and dysbiosis [6]. The results obtained on the effectiveness of probiotics reducing mucosal damage of hyperacidity and *Helicobacter pylori (Hp)*, possibly contributing to limiting the colonisation of the stomach by the bacterium, suggest a possible protective role of yoghurt towards gastric disease [7,8,9,10]. In particular, natural probiotics present in traditional fermented food may counter the damage exerted by Hp on gastric mucosa [10]. The beneficial properties of fermented food for overall and gastrointestinal health have led to the concept of functional food [11].

Dairy products, and yoghurt in particular, are poorly investigated in relation to GC and are often excluded from meta-analyses on nutritional epidemiology [12]. Their properties have been questioned, but scientific evidence showed overall protective effects towards common chronic diseases and cancer, including GC [12]. The few reviews and meta-analyses which addressed this association provided inconsistent results [13,14,15].

The Stomach Cancer Pooling (StoP) Project Consortium [16] provides a unique opportunity to investigate the association between yoghurt intake and GC in a large population of individuals worldwide. We aimed to investigate the role of yoghurt intake on GC risk, including anatomical and histological subtypes, by pooling data from 16 international studies.

## 2. Methods

The present study is based on the StoP Project Consortium (http://www.stop-project.org/ (accessed on 15 February 2023)) [16], which includes 34 case-control or nested-within-cohort studies, forming a total of 13,121 cases and 31,420 controls from 14 countries. The StoP Project aims to examine the role of several lifestyle and genetic determinants in the aetiology of gastric cancer through pooled analyses of individual-level data after central collection and validation of the original datasets. Participating studies were involved through the personal contacts of participating investigators. Principal investigators provided a signed data transfer agreement and, thereafter, the original data set of the study. Two studies (one from Greece and one from Finland) computed their own results locally (through standardised analyses) and then provided estimates for the second-stage meta-analysis to the StoP Project consortium [17,18]. The StoP Project received ethical approval from the University of Milan Review Board (19/15 on 1 April 2015). Detailed information on the overall aims and methods has been described elsewhere [19].

The current analysis is based on 16 studies with information on yoghurt intake, including two studies from Italy [20,21], one from Portugal [22], two from Spain [23,24], two from Greece [7,25], one from Finland [18], one from Japan [26], one from Russia [27], three from Mexico [28,29,30], two from the USA [31,32] and one from Brazil [33]. Of these, nine were hospital-based studies, and seven were population-based studies. Of the latter, three were nested in prospective cohorts. The analysis includes histologically-confirmed GC cases; controls were selected based on hospital or neighbourhood. Additional information on the studies’ characteristics are available in Supplementary Table S1.

Data were harmonised according to a pre-specified format, and completeness and consistency between variables were checked. Yoghurt intake was derived for each study using food frequency questionnaire (FFQ) information. In the original data collection, yoghurt intake was collected either in g per day or portions per day, with country-specific variability (e.g., 1 portion = 125 g in Europe, 150 g in the USA and 200 g in Russia). An overall yoghurt intake variable was generated by summing up the different intakes registered in each study, considering as a measurement unit the number of portions per week. A categorical variable was created considering categories of frequency of yoghurt intake (no intake, >0–0.5, >0.5–1.5, >1.5–4.5, and >4.5 portions per week).

First of all, we run a univariate analysis.

Subsequently, multivariable unconditional logistic regression models were used to estimate the odds ratios (OR) of GC and the corresponding 95% confidence intervals (CI) according to yoghurt intake. The logistic regression models included terms for sex, age (≤55, 56–59, 60–64, 65–69, 70–74, 75+ years), smoking status (never, former, current smoker), socioeconomic status (study-specific low, intermediate, high as defined in each original study based on education, income or occupation), alcohol drinking (never, low: ≤12 g/day, intermediate: 13–47 g/day, high: >47 g/day) and vegetable and fruit intake (low, medium, high defined by study-specific tertiles). A model using the categorical variable for yoghurt was also adjusted for the study centre. This model was fitted on pooled data from 12 studies which shared the full data. An OR estimate for yoghurt intake as a continuous variable was obtained for each of the 16 studies, and then a combined OR was obtained using a random-effect meta-analysis [34]. We chose a two-stage model because of the heterogeneity in the methodology used in the studies included in the pooled analysis. We also performed analyses by anatomical subsite and histological type. Several stratified analyses were run, namely by sex, study design (hospital-based vs. population-based) and geographic region. Moreover, a meta-analysis restricted to the three case-control studies nested in cohorts was also performed, because of the high validity of the results from this type of study.

Also, we performed a sensitivity analysis by adding a term for total calorie intake to the main model. This analysis was restricted to 10 studies with available information on both yoghurt and caloric intake.

The main model was also repeated by using yoghurt intake as a dichotomous variable (no/any weekly intake).

Heterogeneity between strata-specific results was assessed using the Q test.

A *p*-value less than 0.05 was considered statistically significant All the statistical analyses were performed on STATA, version 16.1 (Stata Corp., College Station, TX, USA) [35].

## 3. Results

The analysis included 20,459 subjects, comprising 6278 cases and 14,181 controls. Table 1 shows their distribution by study, sex, age and major covariates. Most of the individuals were of low socioeconomic status (46.9% and 36.9%). Also, cases were more frequently tobacco smokers (23.3% vs. 20.4%) and heavy alcohol drinkers (14.4% vs. 9.8%), while they consumed fewer vegetables and fruits (33.1% vs. 36.1%) than controls. Overall, 21.9% of cases and 9.2% of controls reported a history of GC among first-degree relatives. 

Table 2 presents the results of the main analysis. The univariate analysis showed a significant inverse relationship between increasing portions of yoghurt per week and GC risk (OR = 0.93, 95% CI = 0.91–0.96).

In the adjusted model, the association between yoghurt intake and GC was not linear across categories of intake, and no relationship was found by using yoghurt intake as a continuous variable (OR = 1.00, 95% CI = 0.97–1.03) based on 12 studies. When a dichotomous variable for yoghurt intake was used, we observed an inverse association between any yoghurt intake vs. no yoghurt intake and GC risk (OR = 0.92, 95% CI = 0.85–0.99). The association was similar when the analysis was restricted to the three cohort studies (not shown in detail).

**Figure 1:**
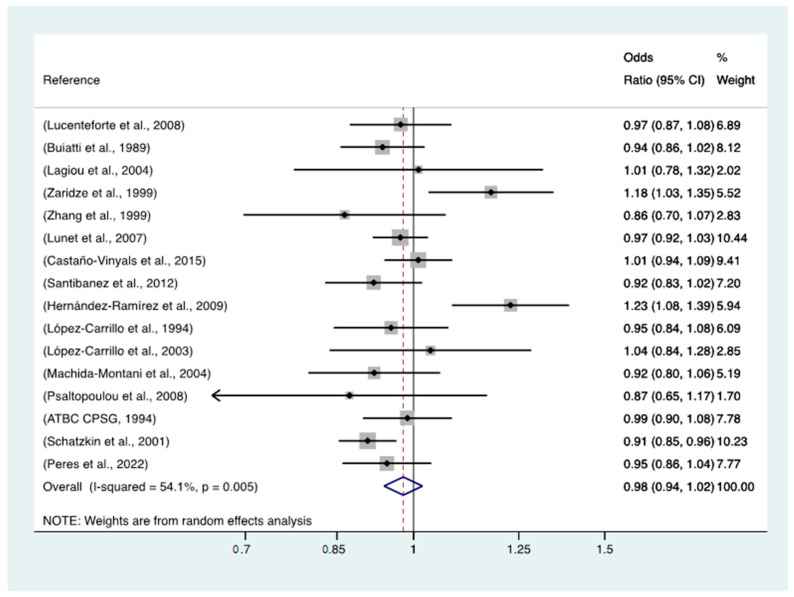
Results of a meta-analysis of the studies included. Notes: ATBC CPSG = The alpha-tocopherol beta carotene Cancer Prevention Study Group. Meta-analysis of the results for portions of yoghurt intake per week is continuous [17,18,20,21,22,23,24,25,26,27,28,29,30,31,32,33].

**Figure 2:**
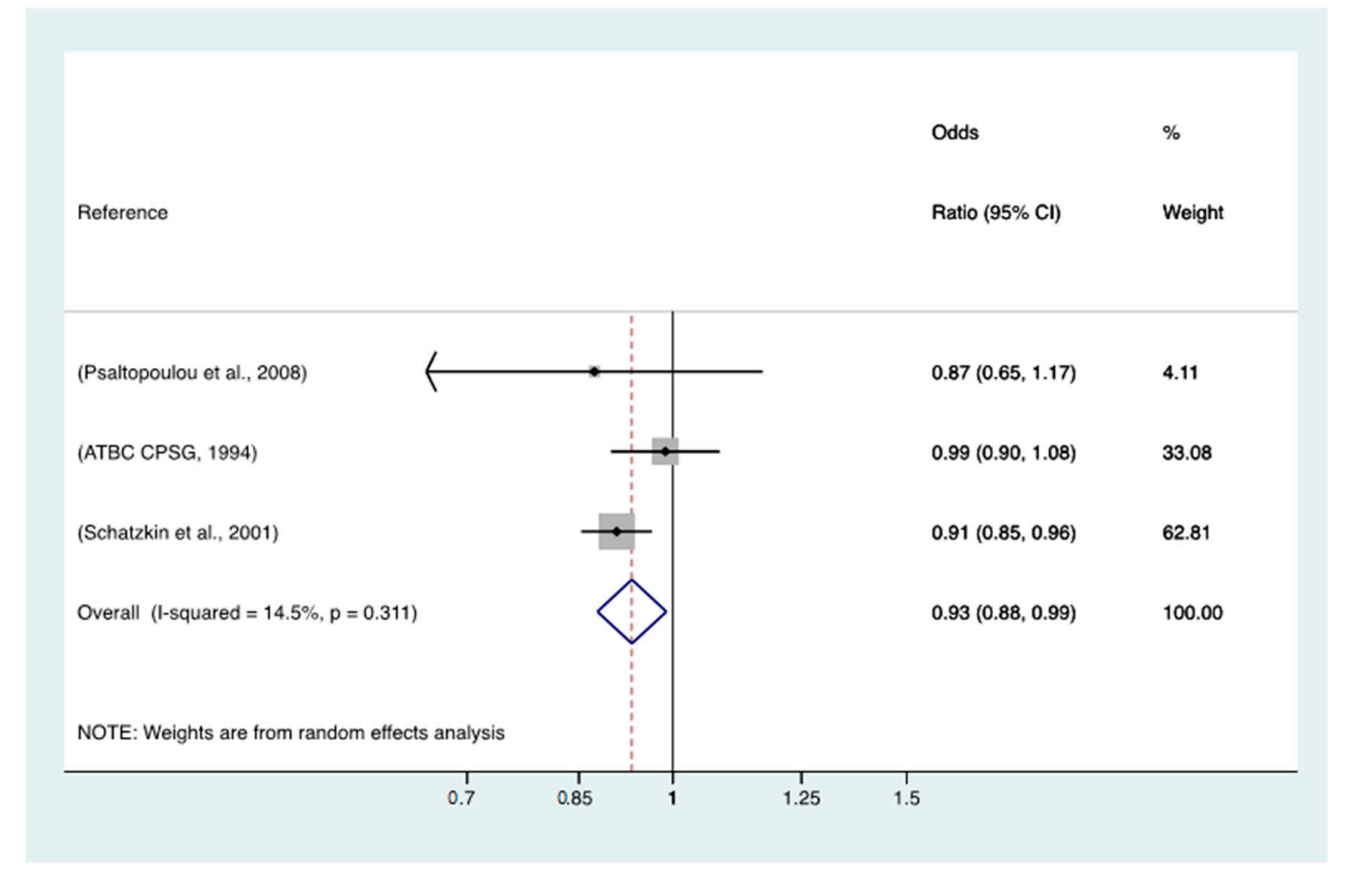
Results of the meta-analysis restricted to the cohort studies included. Notes: ATBC CPSG = The alpha-tocopherol beta carotene Cancer Prevention Study Group. Meta-analysis of the results for portions of yoghurt intake per week is continuous [17,18,32].

The meta-analysis of all 16 studies (Figure 1) revealed a non-significant association between yoghurt intake and GC, with an OR of 0.98 (95% CI = 0.94–1.02, *p* heterogeneity = 0.005) for the highest category of intake vs. no yoghurt consumption. When the meta-analysis was restricted to the three case-control studies nested in cohort studies (Figure 2), a significant inverse relationship was found, with an OR of 0.93 (95% CI = 0.88–0.99, *p* heterogeneity = 0.311).

Table 3 reports the results by GC anatomical subsite and histological type. A linear inverse relationship was suggested for cardia GC (OR = 0.96, 95% CI = 0.90–1.02), while a direct relationship emerged for non-cardia GC (OR = 1.03, 95% CI = 1.00–1.07), and no relationship was identified for the undefined subsite GC cases (OR = 1.02, 95% CI = 0.96–1.08). These results were not significantly heterogeneous (*p* for heterogeneity = 0.15). Also, a significant positive trend was found for diffuse GC when considering an increase in intake of one portion of yoghurt per week (OR = 1.12, 95% CI = 1.07–1.19), no relationship was found with intestinal GC (OR = 1.02, 95% CI = 0.97–1.06), and GC of undefined histology was inversely related to yoghurt consumption (OR = 0.95, 95% CI = 0.91–1.00), with significant heterogeneity among these values (*p* for heterogeneity < 0.001).

Table 4 reports the results of the analysis stratified by study design. No significant association was found in hospital-based as well as population-based studies, without significant heterogeneity (*p* = 0.736).

Additional exploratory analyses by geographic region were limited by the small number of available studies and did not offer further insight into the results.

No effect modification by sex was revealed (*p* = 0.3) (Supplementary Table S2).

No differences were evidenced between the analysis adjusted and unadjusted by caloric intake.

## 4. Discussion

Our pooled analysis of 16 international studies found no association between yoghurt intake and GC risk. Results were consistent across sex, anatomical subsite and histology groups. The analysis restricted to cohort studies showed a moderate inverse relationship between yoghurt intake and GC, while no association was found in hospital-based and population-based case-control studies.

While total yoghurt intake was lower among cases than controls, adjusted analyses from logistic regression models did not find an association between yoghurt intake and GC risk. This is mainly due to unbalances in the case:control ratio across studies with a different mean level of yoghurt intake. In fact, the analysis based on univariate models (only adjusted by the study) showed a significant inverse association between any yoghurt intake and GC risk. It is possible that yoghurt consumption may affect GC risk only at higher intakes than those considered in the present pooled analysis [36]. Energy intake was not a confounder in this study, as demonstrated by the sensitivity analyses performed among 10 studies with the available information. This is consistent with the hypothesis that yoghurt consumers do not present any particular pattern of caloric intake.

Noticeably, when considering yoghurt intake as a dichotomous variable, GC risk resulted in being significantly decreased, which supports the evidence of healthy properties of yoghurt.

There is large variability of locally consumed fermented foods and beverages, which may lead to different effects on human health in different populations. Most traditional fermented foods are natural sources of probiotic microbes, which have been shown to have anti-Hp properties. As pointed out by Nair and coworkers, the geographical difference in GC incidence among countries with a high prevalence of Hp infection (e.g., low GC rates in Africa and India vs. high GC rates in Japan) is not fully explained by the virulence of different Hp strains [10]. The authors hypothesised that the differences in GC epidemiology in these high Hp-risk countries might be partially explained by different patterns of consumption of fermented foods due to the microbial content in ethnic fermented food acting against Hp-induced carcinogenesis [10].

Probiotics have been shown to be effective against different GI diseases, including Hp infection [37]. For example, probiotic supplements may improve the Hp eradication rate [38,39,40]. A study conducted in China on more than 2000 people aged 0 to 77 years found that individuals who reported consuming yoghurt frequently or daily had a lower risk of Hp infection than never or occasional consumers (OR: 0.80, 95% CI: 0.65–1.00), especially restricting the analysis to adults [41]. As reviewed by Scourboutakos et al., yoghurt containing *Lactobacillus casei* DN-114001 is associated with decreased frequency of common respiratory infectious diseases, reduced risk of *Clostridium difficile* and antibiotic-associated diarrhoea in the elderly, and decreased asthma and rhinitis episodes in children [42]. Another bacterial strain, *Bifidobacterium lactis* BB12, was associated with increased gastrointestinal well-being, in particular, reducing abdominal bloating, flatulence and discomfort symptoms [42]. Also, the properties conferred by the combination of different microorganisms have been studied, such as that of *Bifidobacterium lactis* BB12 plus *Lactobacillus acidophilus* LA-5, which, at dosage similar to those of commercial products, improved metabolic parameters (e.g., glycemic control and blood lipids) and antioxidant status in diabetics [42]. The probiotic strain combination has been demonstrated to produce a higher quality yoghurt [43], changing its nutritional composition [43]. For example, *Lactobacillus acidophilus* and *Bifidobacterium bifidum* are associated with higher moisture and low calorie, while *Lactobacillus plantarum* and *Lactobacillus casei* determine higher ash, protein, carbohydrate, energy, calcium and phosphorous content in yoghurt [43]. Also, a different combination of microorganisms acts on the acid and bile salt tolerance, which are important for the survival duration of the live bacteria from yoghurt in the gut [43]. It is important to consider that non-industrial yoghurt may contain different species of bacteria than Lactobacillus and Bifidobacterium, although specific data are scarce [44,45].

Next to the effect conveyed by fermentation of live bacteria, the yoghurt matrix contains other classes of nutrients which may exert a beneficial effect, including vitamins and minerals (e.g., calcium and vitamin D), bioactive fatty acids, and proteins such as whey [46].

Nutritional cancer epidemiology often lacks investigation on dairy products and yoghurt in particular, and available studies show inconsistent results [13,14,15]. A meta-analysis by Sun and coauthors [14] suggested an inverse relationship between yoghurt intake and GC risk despite no significant result, based on three case-control studies and one cohort study (RR = 0.77 for highest vs. lowest yoghurt consumption, 95% CI = 0.58–1.03, *p* for heterogeneity = 0.891 < Egger’s test: *p* = 0.923). The authors found similar results among European and Asian studies and by study design [14]. Another meta-analysis conducted in the same period did not identify any effect of fermented (yoghurt, cheese) and non-fermented (milk) dairy products on GC risk [15]. The research focused on gut microbiota may provide a better understanding of the potential effects of fermented foods such as yoghurt on GC risk [6].

Consistent with our results, a meta-analysis by Guo and coworkers found an inverse association between dairy products intake and GC risk in cohort studies (RR = 0.76 for highest vs. lowest category of total dairy intake 95% CI: 0.64–0.91, based on six cohort studies) but no association in case-control studies [47]. Prospective cohort studies provide more reliable data on lifestyle habits [48]. Moreover, both cohort and case-control studies may fail in reconstructing a comprehensive picture of the dietary habits of the participants, which are largely subjected to changes, and whose effect on health outcomes are mostly seen over the long period [49,50]. The difference we found between the study design (case-control vs. cohort) may reflect the different timing in data collection rather than the different structure of the questionnaires used, namely data collected at baseline vs. data collected at the moment of a cancer diagnosis. No significant difference was found between hospital-based and population-based case-control studies. In this sense, the lack of difference we obtained supports the hypothesis of no relationship between yoghurt and GC.

No specific relationship emerged by anatomical subsite of GC, nor effect modification by sex. This corroborates the hypothesis of the lack of any effect of yoghurt on GC overall. The result we obtained when stratifying by histological type needs to be considered with caution: while a positive association was identified for diffuse GC, an inverse one was observed when considering cases undefined for histological type, suggesting that their redistribution among diffuse and intestinal type would lead to no overall association. It should be considered that data collection on GC epidemiology, including pathology data, is widely heterogeneous among different countries [51] and among different regions of the same country.

We could not identify other studies reporting comparable results for yoghurt intake and anatomical or histological types of GC, limiting the interpretability of the results.

Our pooled analysis has several strengths, starting from a large number of studies included. Notably, by pooling data from the participants of the StoP-Consortium, we could address a topic which has to date been poorly investigated. We could account for several potential confounders, given the detailed information available in the dataset.

This study also suffers from some limitations. The measure we used was the frequency of yoghurt intake, but portion size can vary by country (e.g., one portion was equal to 125 g in most studies but was equal to 200 g in the Russian study). Also, we did not adjust for Hp infection, which is an important risk factor of GC, because of missing data. Further, analyses by anatomical subsite and histological type were impaired by the high proportion of unclassified cases, limiting the interpretability of the results we obtained. In addition, most of the included studies had a case-control design, where questionnaires are administered at the moment of cancer diagnosis among the cases, potentially introducing differential recall bias.

In addition, we could not account for the fact that yoghurt may vary according to the matrix and milk source (e.g., cow or goat). Further, data were too sparse to investigate any difference between different types of yoghurt, including flavoured, frozen, whole milk or low-fat yoghurt. Lastly, while some studies were designed to investigate diet and GC, none were specifically focused on yoghurt. The study was not designed to investigate the role of the type of milk used for yoghurt production (raw, pasteurised, powder), the type and number of microorganisms used in the coagulation process, as well as the time and incubation temperature or additional ingredients (juices, fruits, starch, texturising agents, protein, etc.) that are incorporated during the production steps.

In conclusion, this pooled analysis found no association between yoghurt intake and GC, nor significantly different effects within the anatomical sites or histological types. Cohort studies, as well as analyses treating yoghurt consumption as a dichotomous variable, showed an inverse association between yoghurt intake and GC risk, suggesting that a healthy effect of yoghurt may be evidenced in large prospective studies. The variability in yoghurt composition is an inherent characteristic of this product, which complicates the identification of its effects on human health. Therefore, we recommend caution in interpreting our results, given the lack of information on yoghurt characteristics and composition, which may vary in different populations. The association between yoghurt and GC deserves further investigation, given the potential implication in GC prevention.

## Figures and Tables

**Table 1:** Distribution of cases of gastric cancer and controls according to sex, age and selected covariates.

	Cases [N(%)]	Controls [N(%)]
**Total**	6278 (100.0)	14,181 (100.0)
Sex		
Male	4099 (65.3)	8389 (59.2)
Female	2179 (34.7)	5792 (40.8)
Age (years)		
≤55	1321 (21.1)	3521 (24.8)
56–59	528 (8.4)	1165 (8.2)
60–64	848 (13.5)	1985 (14.0)
65–69	1226 (19.5)	2620 (18.5)
70–74	1326 (21.1)	2584 (18.2)
≥75	1029 (16.4)	2306 (16.3)
Tobacco smoking		
Never	2452 (40.3)	6320 (45.7)
Former	2214 (36.4)	4706 (33.9)
Current	1416 (23.3)	2819 (20.4)
Alcohol drinking		
Never	1591 (26.1)	3835 (28.8)
Low	1721 (28.3)	4725 (35.5)
Intermediate	1899 (31.2)	3441 (25.9)
High	877 (14.4)	1296 (9.8)
Socio-economic status		
Low	2870 (46.9)	5159 (36.9)
Intermediate	2155 (35.2)	5290 (37.8)
High	1100 (17.9)	3530 (25.3)
Vegetables and fruit intake		
Low	1926 (32.0)	3840 (29.7)
Intermediate	1977 (33.9)	4434 (34.3)
High	1934 (33.1)	4673 (36.1)
Gastric cancer subsite		
Cardia	1179 (18.8)	NA
Non-cardia	3463 (55.2)	
Undefined	1198 (19.0)	
Histological type		NA
Intestinal	1717 (27.3)	
Diffuse	1191 (18.9)	
Undefined	2249 (35.8)	
Mean number of portions of yoghurt per week	0.16 (0.15–0.17)	0.25 (0.24–0.26)

**Table 2:** Odds ratios (OR) and 95% confidence intervals (CI) of the association between portions of yoghurt intake per week and gastric cancer (GC), based on the study [21,22,23,24,25,26,27,28,29,31,32,33].

Exposure	Cases-Controls	Overall GC OR (95% CI) Adjusted Model	Overall GC OR (95% CI) Raw Model
Portions of yoghurt intake per week			
0	3103–5552	Ref	Ref
>0–0.5	1259–2705	0.86 (0.78–0.95)	0.79 (0.72–0.87)
>0.5–1.5	539–1417	0.96 (0.85–1.08)	0.79 (0.70–0.88)
>1.5–4.5	470–1650	0.86 (0.76–0.98)	0.71 (0.63–0.80)
>4.5	535–2020	1.08 (0.95–1.22)	0.84 (0.75–0.95)
Continuous (1 category per week increase)		0.93 (0.97–1.03)	0.93 (0.91–0.96)
No/Any yoghurt intake	0.92 (0.85–0.99)		0.78 (0.73–0.84)

Notes: The adjusted model included study, sex, age, tobacco smoking, alcohol drinking, socioeconomic status and fruit and vegetable intake.

**Table 3:** Odds ratios (OR) and 95% confidence intervals (CI) of the association of yoghurt intake by approximate portions of yoghurt intake and GC subsites and histological types.

Exposure	Subsite of GC			Histology of GC		
	Cardia (No = 1179) OR (95%CI)	Non-Cardia (No = 3463) OR (95%CI)	Undefined (No = 1198) OR (95% CI)	Diffuse (No = 1191) OR (95%CI)	Intestinal (No = 1717) OR (95%CI)	Undefined (No = 2249) OR (95% CI)
Portions of yoghurt intake per week						
0	Ref	Ref	Ref	Ref	Ref	Ref
>0–0.5	0.96 (0.81–1.13)	0.95 (0.83–1.09)	0.93 (0.78–1.11)	1.05 (0.83–1.33)	0.89 (0.72–1.11)	0.87 (0.76–0.99)
>0.5–1.5	0.96 (0.74–1.24)	1.01 (0.87–1.17)	1.11 (0.89–1.40)	1.34 (1.06–1.68)	0.88 (0.71–1.09)	0.92 (0.75–1.13)
>1.5–4.5	0.98 (0.75–1.29)	0.93 (0.79–1.09)	0.97 (0.76–1.25)	1.15 (0.90–1.48)	0.90 (0.71–1.13)	0.88 (0.69–1.12)
>4.5	0.79 (0.58–1.06)	1.23 (1.06–1.42)	1.11 (0.85–1.45)	1.73 (1.38–2.18)	1.18 (0.96–1.45)	0.83 (0.65–1.08)
Continuous (1 category increase)	0.96 (0.90–1.02)	1.03 (1.00–1.07)	1.02 (0.96–1.08)	1.12 (1.07–1.19)	1.02 (0.97–1.06)	0.95 (0.91–1.00)
*p* heterogeneity	*p* = 0.15			*p* = <0.001		

Notes: The model included study, sex, age, tobacco smoking, alcohol drinking, socioeconomic status, and fruit and vegetable intake. *p* for heterogeneity is calculated on the continuous variable. No = number. Cardia GC is available for [20,21,22,23,24,26,27,31,32,33]. Non-cardia GC is available for [20,21,22,23,24,25,26,27,28,31,32,33]. Undefined GC subsite is available for [20,22,23,24,25,26,27,28,31,32,33]. Diffuse and intestinal GC is available for [20,21,22,23,24,27,28,31,32,33]. Undefined GC histology is available for [20,21,22,23,24,27,28,31,32,33].

**Table 4:** Odds ratios (OR) and 95% confidence intervals (CI) of the association of yoghurt intake by approximate portions of yoghurt intake and study design.

Exposure	Study Design	
	Case-Control Hospital-Based OR (95%CI)	Case-Control Population-Based OR (95% CI)
Yoghurt intake		
0	Ref	Ref
>0–0.5	0.78 (0.64–0.95)	0.86 (0.78–0.95)
>0.5–1.5	0.97 (0.79–1.21)	0.96 (0.85–1.08)
>1.5–4.5	0.81 (0.65–1.02)	0.86 (0.76–0.98)
>4.5	1.13 (0.89–1.44)	1.08 (0.96–1.23)
Continuous (1 category increase)	1.01 (0.96–1.06)	1.00 (0.97–1.03)
*p* heterogeneity	0.736	

## Data Availability

Data can be obtained from the StoP Project according to the provisions set up in the Consortium (http://stop-project.org/, accessed on 5 December 2022). Further information is available from the corresponding author upon request.

## Supplementary Materials

The following supporting information can be downloaded at: https://www.mdpi.com/article/10.3390/nu15081877/s1, Table S1: Characteristics of StoP Consortium Studies. Table S2: Results of the analysis stratified by sex.
