# Supplementary material for: Yoghurt Intake and Gastric Cancer: A Pooled Analysis of 16 Studies of the StoP Consortium
Source: Nutrients. 2023 Apr 13;15(8):1877. doi: 10.3390/nu15081877 (PMC10147010; doi:10.3390/nu15081877)
Supplement: Supplementary file 1 [file nutrients-15-01877-s001.zip › nutrients-2267886-supplementary.pdf]

nutrients

MDPI

Supplementary Table S1. Characteristics of StoP Consortium Studies.

Study Name	Country	Reference	Study Period	Cases (n)	Control (n)	Control Type	Yoghurt Data
Milan, Italy [20]	Milan, Italy	Lucenteforte et al., 2008	1997–2007	230	547	Hospital-based	1 portion= 125 g; weekly intake
Multicenter, Italy [21]	4 areas, Italy	Buiatti et al., 1989	1985–1987	1016	1159	Population-based	Weekly intake in the previous 2/3 years
Athens, Greece [25]	Greece	Lagiou et al., 2004	1981–1984	110	100	Hospital-based	Weekly intake
Moscow, Russia [27]	Russia	Zaridze et al., 1999	1996–1997	450	611	Hospital-based	1 portion = 200 g; weekly intake
New York, USA [31]	USA	Zhang et al. 1999	1992–1994	132	132	Hospital-based	Flavored yoghurt; 1 cup; frequency of consumption
Porto, Portugal [22]	Portugal	Lunet et al., 2007	1999–2006	692	1667	Population-based	1 portion = 125 g; frequency of consumption
10 provinces, Spain [23]	Spain	Castano-Vinyals et al., 2015	2008–2012	441	3440	Population-based	Whole and skimmed yoghurt; frequency of consumption
Valenica, Spain [24]	Spain	Santibanez et al., 2012	1995–1999	401	455	Hospital-based	1 portion = 125 g; frequency of consumption
Mexico City 1, Mexico [28]	Mexico	Hernandez -Ramirez et al., 2009	2004–2005	248	478	Population-based	NA
Mexico City 2, Mexico [29]	Mexico	Lopez-Carrillo et al., 1994	1989–1990	220	752	Population-based	NA
3 areas, Mexico [30]	Mexico	Lopez-Carrillo et al., 2003	1994–1996	234	468	Hospital-based	NA
Nagano, Japan [26]	Japan	Machida-Montani et al., 2004	1998–2002	153	303	Hospital-based	Frequency of consumption
Greece [17]	Greece	Psaltopoulou et al., 2008	1994–1999	82	410	Cohort, nested case-control (EPIC-GREECE study)	NA
Finland [18]	Finland	The ATBC Cancer Prevention Study Group	1985–1988	486	972	Cohort, nested case-control (ATBC study)	NA
6 States, USA [32]	USA	Schatzkin et al., 2001	1995–1996	1583	3331	Cohort, nested case-control (AARP study)	Frequency of consumption
Brazil [33]	Brazil, multicentric	Peres SV et al., 2022	2016–2019	368	738	Hospital-based	Frequency of consumption

Notes: ATBC = Alpha-Tocopherol, Beta-Carotene.

Supplementary Table S2. Results of the analysis stratified by sex.

Sex Subgroup	Yoghurt Intake OR (95% CI)				
	Q1	Q2	Q3	Q4	Q5
Male	Ref	0.89 (0.79–1.01)	0.89 (0.75–1.04)	0.86 (0.72–1.02)	1.08 (0.91–1.28)
Female	Ref	0.82 (0.69–0.98)	1.03 (0.84–1.25)	0.89 (0.73–1.09)	1.15 (0.95–1.40)
Continuous					
Male			0.99 (0.96–1.03)		
Female			1.02 (0.98–1.07)		
P heterogeneity			0.3		
